# 

**Published:** 1910-12-03

**Authors:** 


### CONTENTS.

| | PAGE |
|---|---|
| **Unqualified Medical Practice** ... ... ... ... ... ... | 275 |
| **The Problems of Nephritis** ... ... ... ... ... ... | 276 |
| **Annotations—** | |
| The Consumption Crusade—Smallpox in South America—Mr. Schröder's Deputy ... ... ... ... ... ... | 277 |
| **Medical Opinion and Movement** ... ... ... ... | 278 |
| **Hospital Clinics—** | |
| Insects as Carriers of Tropical Diseases. By G. C. Low, M.D. | 281 |
| **Medicine—** | |
| Galvanic Treatment for Comedones ... ... ... ... ... | 284 |
| **Special Article—** | |
| Death and Dying in Plastic and Pictorial Art ... ... ... | 285 |
| **Practical Notes on Diagnosis and Treatment** ... | 286 |
| **The General Practitioner's Column—** | |
| Medicated Squab Pie ... ... ... ... ... ... | 287 |
| A Simple and Efficient Method of Removing Tonsils ... ... | 288 |
| **Laryngology and Rhinology—** | |
| Tuberculosis of the Air-passages above the Larynx ... ... | 289 |
| **Bacteriology—** | |
| The Bacillus Influenzæ ... ... ... ... ... ... | 290 |
| **Tropical Medicine—** | |
| Human Botryomycosis ... ... ... ... ... ... ... | 290 |
| **News and Events of the Week** ... ... ... ... ... | 291 |
| **Obituary** ... ... ... ... ... ... ... ... ... ... | 292 |
| **Appointments and Resignations** ... ... ... ... | 292 |
| **Parliament and Publications** ... ... ... ... ... | 292 |
| **The Week's Work—** | |
| Eminent Chairman Series—A New Medical Superintendent for the Hope Hospital, Salford—The Park Hospital for Children—The Largest Children's Hospital in the World—What the Park Hospital Means—Hospitals in the Cape State—A Hospital as Successful Litigant—This Week's Drug Market ... ... ... ... ... ... ... ... | 293 |
| **Special Institutional Articles—** | |
| The Need of an Intermediate Single-room Service in Hospitals | 295 |
| The Park Hospital for Sick Children... ... ... ... ... | 297 |
| Hospital Medical Schools and the King's Fund ... ... ... | 299 |
| **Jottings** ... ... ... ... ... ... ... ... ... ... | 300 |
| **Institutional Notes and News** ... ... ... ... ... | 301 |
| **Gifts and Bequests** ... ... ... ... ... ... ... | 3 2 |
| **The Hospital World** ... ... ... ... ... ... ... | 303 |
| **Metropolitan Hospital Sunday Fund** ... ... ... | 304 |

